# Follicular Growth Evaluation, Oviposition and Egg Hatching in the Ball Python (*Python regius*)

**DOI:** 10.3390/ani16162555

**Published:** 2026-08-16

**Authors:** Alessandra Rota, Michele Lorini, Francesco De Filippo, Cristiana Manetti, Matteo Tesi

**Affiliations:** 1 Dipartimento di Scienze Veterinarie, Università degli Studi di Pisa, 56124 Pisa, Italy; lorinimic@gmail.com (M.L.); manetti.c@hotmail.com (C.M.); matteo.tesi@unipi.it (M.T.); 2 Exovet Team, 80014 Giugliano in Campania, Italy; francesco.defilippo@pec.vetnapoli.it

**Keywords:** *Python regius*, reptile, reproduction, follicular growth, ultrasound, breeding management

## Abstract

Reproductive performance and follicular dynamics in captive female ball pythons (*Python regius*) were investigated to evaluate reproductive efficiency, assess the usefulness of ultrasonography, and examine the relationship between follicular size and ovulation timing. Reproductive parameters included follicular development, ovulation rate, ovulation-to-oviposition interval, egg quality, and hatching success. Monthly ultrasonographic examinations were performed from March to August using a 7.5 MHz linear probe, and the findings were compared with manual palpation. Overall, 58.3% of females ovulated and laid eggs. Ovulation occurred mainly between April and August, whereas egg laying took place from June to November. The median clutch size was six eggs, and incubation lasted approximately 58 days. Ultrasonography effectively monitored follicular development, showing progressive changes in echogenicity and significant increases in follicular diameter throughout the breeding season. A strong negative correlation was found between follicular size and days to ovulation (r = −0.88; *p* < 0.001). Follicles measuring ≥20 mm showed a median growth rate of 0.38 mm/day. Manual palpation showed lower diagnostic accuracy, with a 25% false-negative rate. This study provides baseline reproductive data that may improve captive breeding management, animal welfare, and future reproductive research, including the development of reproductive biotechnologies in this species.

## Simple Summary

Successful reproduction is essential for the sustainable management of captive ball python (*Python regius*) populations, yet information on reproductive monitoring and efficiency in this species remains limited. This study was conducted to evaluate reproductive performance in captive females and investigate the utility of ultrasonography for assessing follicular development and predicting ovulation. Females were monitored throughout the breeding season, and reproductive outcomes were compared with ultrasonographic findings. Ultrasonography proved to be a reliable and minimally invasive method for tracking follicular growth, estimating the proximity of ovulation and outperforming manual palpation. Follicular size was associated with ovulation timing, providing a useful indicator of reproductive status. The data collected may be valuable for evaluating and improving breeding management, reproductive success, and welfare in captive ball pythons.

## 1. Introduction

The ball python (*Python regius*) is a snake native to West and Central Africa, inhabiting both savannah and rainforest ecosystems. The climate in these regions is typical of tropical sub-Saharan environments, characterized by distinct dry and wet seasons and relatively limited monthly variation in maximum and minimum temperatures. In general, pythons, particularly females, occupy small burrows, often after preying upon the small mammals that originally constructed them. Like many other snake species, pythons play an important role in ecosystem health and functioning, as well as in supporting local livelihoods in their native range. In several countries (e.g., Togo, Ghana, Benin) “snake hunters” have collected ball pythons from the wild to rear in farms or for export [1]. In 2010, the ball python was classified as “Least Concern” on the IUCN Red List of Threatened Species; however, by 2022, it had been reclassified as “Near Threatened” [2].

*Python regius* has become a popular exotic pet, largely due to its relatively small size (1.5–1.8 m), docile temperament, low maintenance costs, modest husbandry requirements, limited space needs, and the wide variety of available morphs. The price of juvenile animals can be as low as a few tens of USD, making them widely accessible, although rare morphs may reach considerably higher prices, up to USD 30,000 or more [3]. Consequently, ball python breeding can be both creative and profitable. Nevertheless, improvements in trade and breeding conditions, particularly with respect to housing, environmental enrichment, substrate, hygiene, and overall health management, are needed [4], as these factors may also enhance reproductive efficiency. Given that ball pythons are ectothermic, maintaining appropriate environmental temperature and humidity is essential for their health in captivity. In contrast, the effects of other husbandry-related factors such as housing, mobility, hygiene, and general health status on reproductive activity remain less well understood. Overall, studies on python reproduction in captivity are still limited [5,6,7,8,9].

Reproductive management in this species includes monitoring follicular development, planning pairings, detecting ovulation and oviposition, incubating eggs, and overseeing hatching. Ultrasonography is a key diagnostic tool in veterinary medicine, widely used to assess multiple organs and systems, including the reproductive tract, in both conventional and non-conventional animal species. In snake medicine, the use of ultrasonography has been described for evaluating coelomic organs and other structures [10,11,12] or for sex determination [13,14]. Despite its potential utility, its application in reptile reproductive management remains relatively underreported. In ball pythons, ultrasonography has been used to evaluate follicular growth before ovulation and estimate clutch size prior to oviposition, although these parameters are still more commonly assessed through coelomic palpation [6,7,8]. In a recent survey, most respondents (93.5%) did not prescreen females for follicle presence before pairing [15].

The aims of this study were: (1) to determine the proportion of ovulations, the number and quality of eggs laid, and the number of successful hatchings among mated female ball pythons (*Python regius*); (2) to describe the size and growth of ovarian follicles, as measured by ultrasonography during the study period; (3) to assess the relationship between follicular size and the time of ovulation; and (4) to compare follicular diameters obtained via ultrasonography with those estimated through manual palpation.

## 2. Materials and Methods

### 2.1. Animals and Husbandry

A total of 48 adult female ball pythons (*Python regius*) were included in the study: 16 from breeding facility 1 (BF-1) and 32 from breeding facility 2 (BF-2), both located in the Naples area (Italy). Animals were housed in plastic containers (80 × 60 × 20 cm) arranged in rack systems. Each container was equipped with a heating mat providing a constant temperature of 32 °C at the warm spot. During the period preceding ovulation, a cooler temperature cycle was applied: 32 °C from 08:00 to 20:00 and 26 °C from 20:00 to 08:00. Relative humidity was maintained at approximately 80%. Substrate differed between facilities: BF-1 used de-resinated spruce shavings (SAFE, Rosenberg, Germany), whereas BF-2 used dried, dust-free beech shavings (Avipur, Sitta S.r.l., San Daniele del Friuli, Italy). In both facilities, the substrate was replaced regularly. The diet consisted of thawed rats (100–150 g), offered every two weeks in BF-1 and every three weeks in BF-2. Females were approximately 3 years old and weighed at least 1.3 kg at the beginning of the study. Each individual was identified based on its morph.

### 2.2. Ultrasonographic Examination

During the 2024 breeding season, the reproductive tract of all animals was monitored using ultrasonography (Mindray DP10 Vet, Shenzhen, China) equipped with a 7.5 MHz linear probe to assess follicular dynamics. Examinations were performed approximately once per month, starting on 2 March 2024, and continued until ovulation or, in the absence of ovulation, until 18 August 2024. All ultrasound examinations were performed by the same operator (FDF) to minimize operator-dependent variability [7]. For each examination, females were removed from their enclosure and placed in a plastic box (80 × 30 × 15 cm). Animals were generally examined without restraint or sedation; in more active individuals, minimal manual restraint was applied to prevent escape. Ultrasound gel was applied to improve image quality and was removed after the examination, before returning the animal to its enclosure. Scans were performed through short lateral approaches targeting the posterior third of the body. The probe was moved in cranio-caudal and dorso-ventral directions using gentle oscillating movements. Each examination lasted approximately 30 s and never exceeded one minute, minimizing handling stress. At each session, the maximum diameter of the largest follicle was recorded.

### 2.3. Ovarian Palpation

Ovarian palpation was performed in a subset of 16 females from BF-1 by two independent operators. The aim was to detect the presence and estimate the diameter of the largest follicle. Each operator recorded their measurements independently prior to ultrasonographic evaluation. Assessments were conducted at three time points (2 March, 23 March and 29 April 2024). Palpation was performed manually by allowing the snake to move toward a plastic box while gently but firmly holding the distal half of the body without applying excessive pressure. Follicles were identified as firm, spherical structures.

### 2.4. Ovulation Assessment

Ovulation was identified based on the appearance of a marked increase in body volume in the caudal third, persisting for several days, the perception of a watery mass upon palpation, and the appearance of a ventral convexity [16,17].

### 2.5. Breeding Management

Females were paired with males for 1–2 days every two weeks, starting approximately 20 days after the initiation of the temperature cycling protocol or when follicles reached an estimated diameter of ~20 mm and continuing until ovulation. Following ovulation, females were monitored daily. Immediately after oviposition, eggs were collected, assessed for the presence of infertile eggs (“slugs”), separated in a different box for each clutch, and incubated in a custom-built incubator at 31.5 ± 0.5 °C and 80–90% relative humidity until hatching.

### 2.6. Statistical Analysis

The distribution of continuous variables was assessed using the Shapiro–Wilk test and data were presented either as mean ± standard deviation or as median and interquartile range for normal or non-normal distribution, respectively. Differences between BF-1 and BF-2 in the proportion of ovulating females and egg-laying rates were evaluated using Fisher’s exact test. Differences in ovulation and egg-laying dates were assessed using the Mann–Whitney U test. Differences in follicular diameter between females that subsequently ovulated and those that did not were evaluated using the Kruskal–Wallis test. Changes in follicular diameter over time within the same individuals were analyzed using the Wilcoxon matched-pairs test. Differences between BF-1 and BF-2 in ovulation-to-laying interval, clutch size, proportion of infertile eggs (“slugs”), incubation duration, and number of live hatchlings were assessed using either Student’s *t*-test or the Mann–Whitney U test, depending on data distribution. The relationship between follicular diameter on 23 March and days to ovulation was analyzed using Spearman’s correlation. For receiver operating characteristic (ROC) curve analysis, the individual female was the unit of analysis. Only females that ovulated and for which the ovulation date was recorded were included. Each female contributed a single observation, defined as the maximum follicular diameter measured at the last ultrasonographic examination before the clinically recorded ovulation. The 30-day prediction window was anchored to the date of this selected examination. Observations were obtained from the examinations performed on 23 March 2024 (*n* = 9), 29 April 2024 (*n* = 6), and 1 June 2024 (*n* = 7). The binary outcome was defined as ovulation occurring within 30 days versus more than 30 days after the selected examination. The optimal threshold was identified using Youden’s index. The 95% confidence interval for the area under the curve (AUC) was estimated using DeLong’s method. For clinical applicability, the optimal threshold was rounded to the nearest whole millimeter, and sensitivity, specificity, positive predictive value, negative predictive value, and accuracy were calculated with exact Clopper–Pearson 95% confidence intervals. The threshold and its diagnostic performance were derived and evaluated in the same dataset, without internal validation. Correlation between palpation and ultrasonographic measurements, as well as between the two operators, was assessed using Spearman’s correlation coefficient. Agreement between palpation and ultrasonography was further evaluated using Bland–Altman plots, and differences between methods were tested using the Wilcoxon matched-pairs test. Statistical analyses were performed using Microsoft Excel, Minitab^®^ 21.4.2 (64-bit) (Minitab LLC, State College, PA, USA), and jamovi version 2.6.44.0 (Jamovi project team, Sydney, Australia). ROC curve analysis was performed using R Statistical Software version 4.6.1 (R Foundation, Vienna, Austria) and the pROC package version 1.19.0.1.

## 3. Results

The overall proportion of females that ovulated and laid eggs was 58.3% (28/48), with similar values observed in BF-1 (9/16, 56.3%) and BF-2 (19/32, 59.4%) (*p* > 0.05). Ovulation dates were not recorded in 6 out of 28 cases. In the remaining 22 animals, the mean ovulation date was 22 May (range: 14 April–8 August). Median ovulation dates were 15 May in BF-1 and 16 May in BF-2 (*p* > 0.05). Egg-laying dates were recorded for all 28 females, with a mean date of 12 July (range: 2 June–10 November). Specifically, oviposition occurred in June (*n* = 13), July (*n* = 5), August (*n* = 7), and November (*n* = 1). Median egg-laying dates were 7 July in BF-1 and 17 June in BF-2 (*p* > 0.05).

Data on the ovulation-to-laying interval, clutch size, proportion of infertile eggs (“slugs”), incubation duration, and neonatal outcomes are summarized in Table 1.

The ovulation-to-laying intervals differed between facilities, ranging from 45 to 62 days in BF-1 and from 35 to 59 days in BF-2. The proportion of “slugs” also differed: in BF-1, only one infertile egg was observed across nine clutches, whereas in BF-2, slugs were present in 7 out of 19 clutches, with two cases affecting 100% of the eggs.

**Figure 1:**
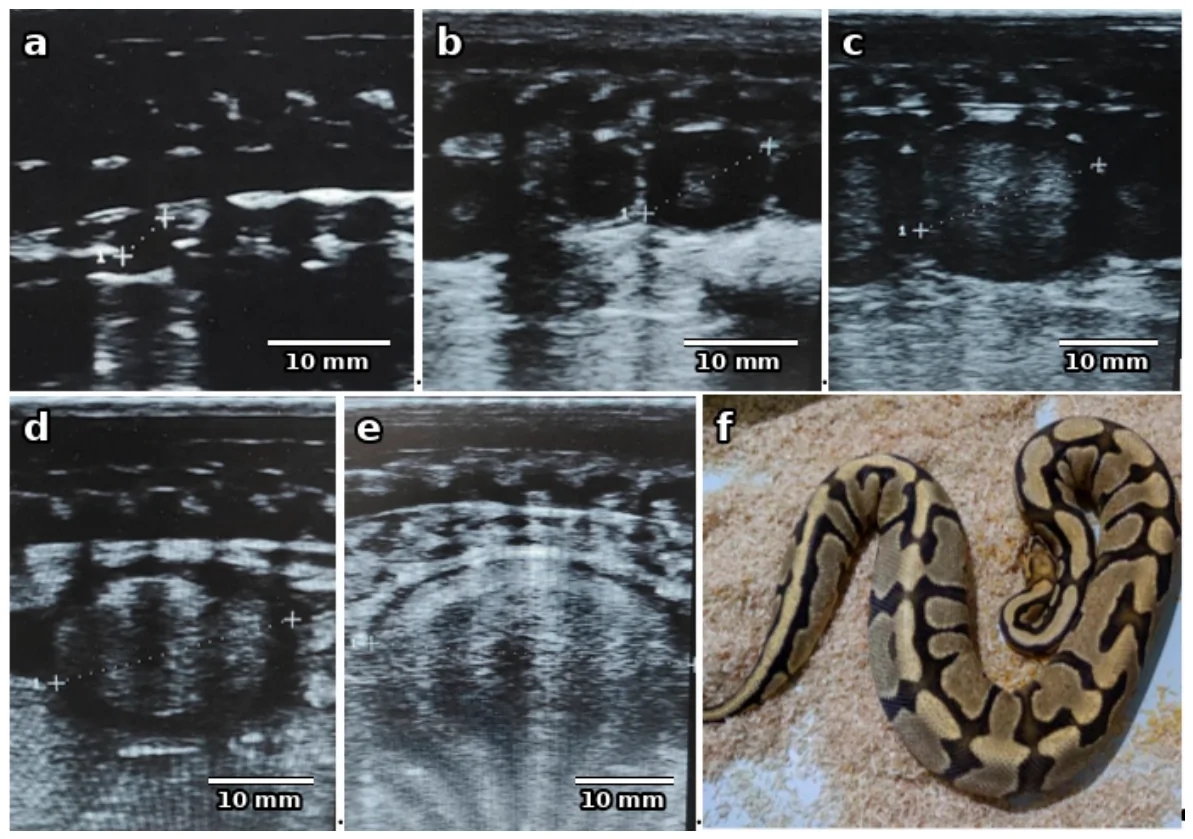
Ultrasound images of (**a**) a chain of follicles, the largest measuring 4.8 mm; (**b**) a 12.5 mm follicle; increased echogenicity begins to become visible in its central area; (**c**) a 19.6 mm follicle; the echogenicity is markedly increased and the shape is still round; (**d**) a 23.3 mm follicle, with a slight vertical flattening; (**e**) a 34.4 mm diameter, oval-shaped follicle; (**f**) ovulation and body swelling.

Follicles were readily identified by ultrasonography and exhibited features consistent with previous reports [7,8]. Initially, follicles appeared anechoic; as their diameter increased, the central region became progressively more echogenic and the shape changed from round to oval (Figure 1).

At the first ultrasound examination (2 March), the median diameter of the largest follicle in the 48 females was 12.4 mm (Q1: 9.5; Q3: 15.9; range: 4.8–26.1 mm). At the second examination (23 March), no females had ovulated, and the median diameter increased to 14.7 mm (Q1: 11.0; Q3: 21.8; range: 5.7–34.4 mm).

The increase in follicular diameter between the first and second examinations was statistically significant (*p* < 0.001), with a mean increase of 3.6 ± 4.2 mm (range: −2.0 to 11.7 mm). However, in 13 out of 48 females (27.1%), no increase was observed; only four of these subsequently ovulated.

At the third evaluation (29 April), 10 females had already ovulated and were excluded from ultrasound examination. Among the remaining 38 females, the median follicular diameter was 14.6 mm (Q1: 10.3; Q3: 23.1; range: 5.5–39.0 mm). The increase from the second examination was significant (*p* = 0.001), with a mean increase of 4.3 ± 6.8 mm (range: −5.2 to 24.4 mm). In 14 out of 38 females (36.8%), no increase in follicular diameter was observed, and none of these subsequently ovulated.

At the fourth evaluation (1 June), a further seven females had ovulated. Among the remaining 31 females, the median follicular diameter was 14.0 mm (Q1: 10.8; Q3: 26.4; range: 5.0–39.3 mm). The increase between the third and fourth examinations was significant (*p* < 0.001), with a mean increase of 4.4 ± 5.4 mm (range: −3.5 to 15.5 mm). In 6 out of 31 females (19.3%), no increase was observed, and none of these subsequently ovulated.

At the fifth evaluation (20 July), nine additional females had ovulated. One female that ovulated on August 8 was not examined, as were two individuals without measurable follicles at the subsequent session. In the remaining 19 females, the median follicular diameter was 10.5 mm (Q1: 7.15; Q3: 14.8; range: 5.0–23.0 mm). In 8 out of 19 females (42.1%), no increase in follicular diameter was observed, and none of these ovulated by the end of the study.

At the final evaluation (18 August), one additional ovulation was recorded. In 18 out of 21 females (85.7%), no increase in follicular diameter was observed. In 13 of these, no measurable follicles were present (recorded as 0 mm), resulting in a median diameter of 0.0 mm (Q1: 0.0; Q3: 8.25; range: 0.0–25.0 mm). The female with the largest follicle (25 mm) laid eggs on November 10.

Throughout the study, only two females that showed follicles larger than 20 mm (20.2 and 22.7 mm in July) did not ovulate; in both cases, follicular diameter decreased at the subsequent examination. The largest follicular diameters observed in the six ultrasound sessions in females that subsequently ovulated and in those that did not during the study are described in Table 2.

**Figure 2:**
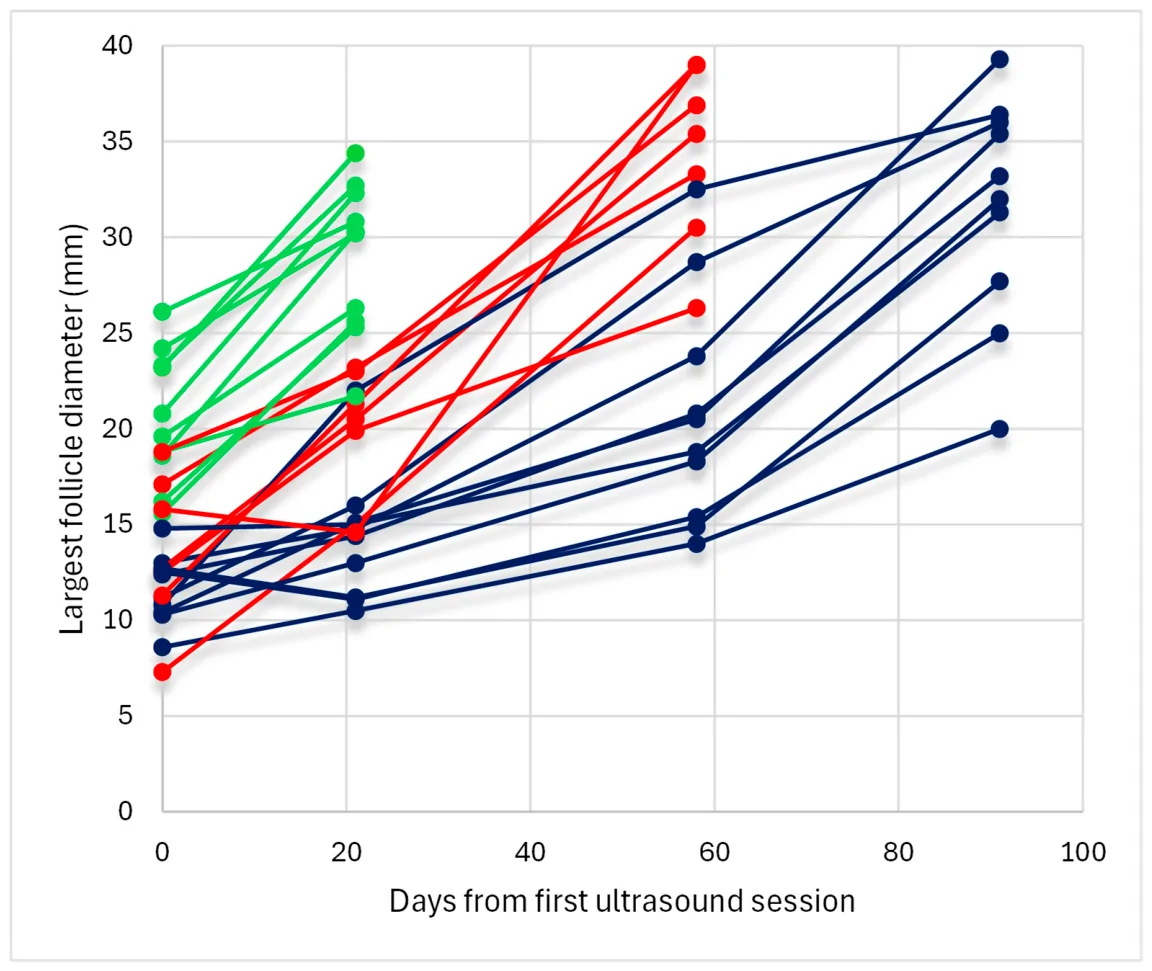
Changes in the diameter of the largest follicle from 2 March to 1 June in 27 female ball pythons that ovulated during the study. Green lines: ovulation between 23 March and 29 April; Red lines: ovulation between 29 April and 1 June; Blue lines: ovulation after 1 June. For graph clarity, the female ovulating after 18 August is not included. Her largest follicle diameters were 15.4, 15.1, 15.8, 20.0, 23.0 and 25 mm at the six ultrasonographic evaluations, respectively.

**Figure 3:**
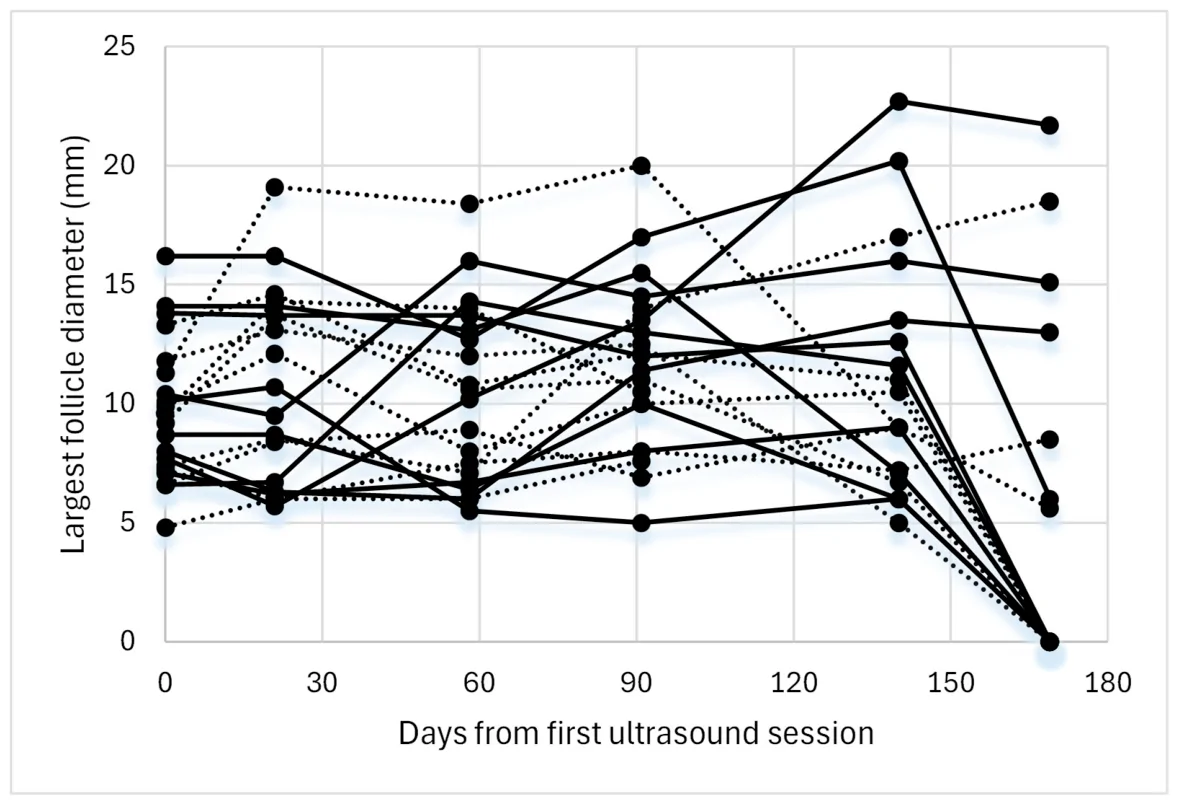
Evolution of largest follicle diameters in 20 non-ovulating female ball pythons from 2 March (day 0) to 18 August. Dotted lines indicate females whose follicular diameter increased between the first and the second ultrasound session.

In females that subsequently ovulated, follicles measuring ≥20 mm showed a median daily growth rate of 0.38 mm/day (IQR 0.25–0.46; range 0.07–0.55 mm/day; *n* = 15 females). The follicular development of ovulating and non-ovulating females is shown at the individual level in Figure 2 and Figure 3.

**Figure 4:**
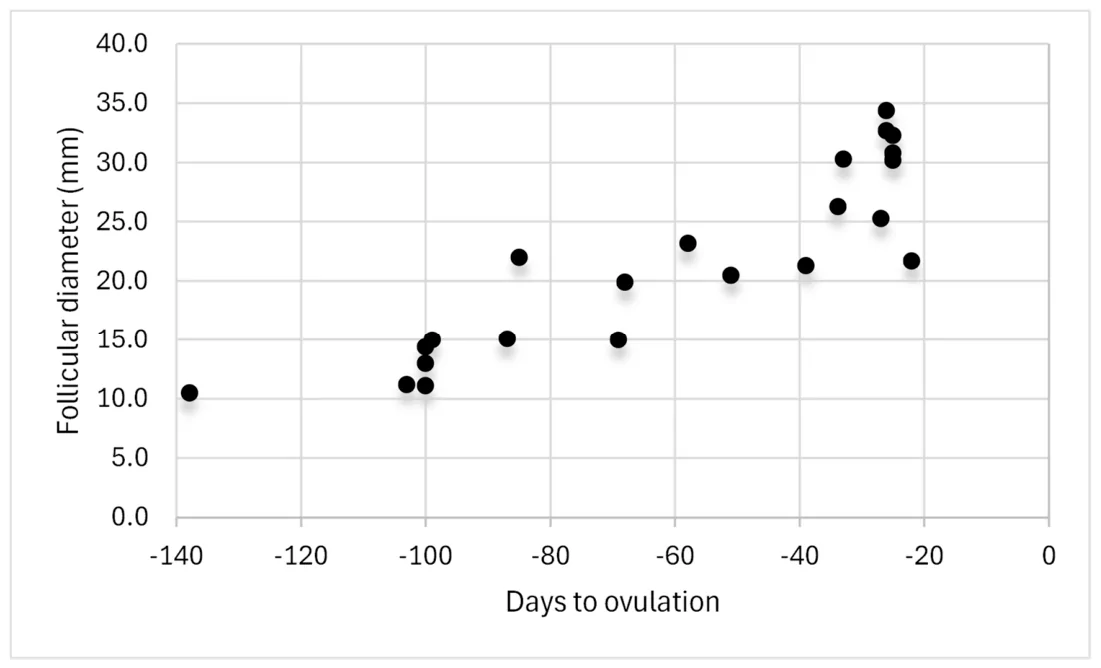
Scatter plot of follicular diameter versus days to ovulation (r = 0.88; *p* < 0.001).

A strong negative correlation was observed between follicular diameter and days to ovulation (r = −0.88; *p* < 0.001, Figure 4).

ROC analysis included 22 females, each contributing one observation. Ovulation occurred within 30 days of the selected ultrasonographic examination in 16 females and more than 30 days after the examination in six females. The area under the ROC curve was 0.82 (95% CI: 0.64–1.00). The optimal follicular diameter threshold was 30.65 mm. For practical clinical application, this threshold was rounded to ≥31 mm. At this cut-off, sensitivity was 68.8% (95% CI: 41.3–89.0%), specificity was 100% (95% CI: 54.1–100%), positive predictive value was 100% (95% CI: 71.5–100%), negative predictive value was 54.5% (95% CI: 23.4–83.3%), and overall accuracy was 77.3% (95% CI: 54.6–92.2%).

Regarding palpation, 45 paired palpation–ultrasound evaluations were performed on 16 females during the first three sessions. Operator 1 estimated follicular diameter in 30 cases, and Operator 2 in 26 cases. Unsuccessful assessments were attributed to the presence of gas (*n* = 1), ingesta (*n* = 1), or difficulty distinguishing small follicles from feces.

**Figure 5:**
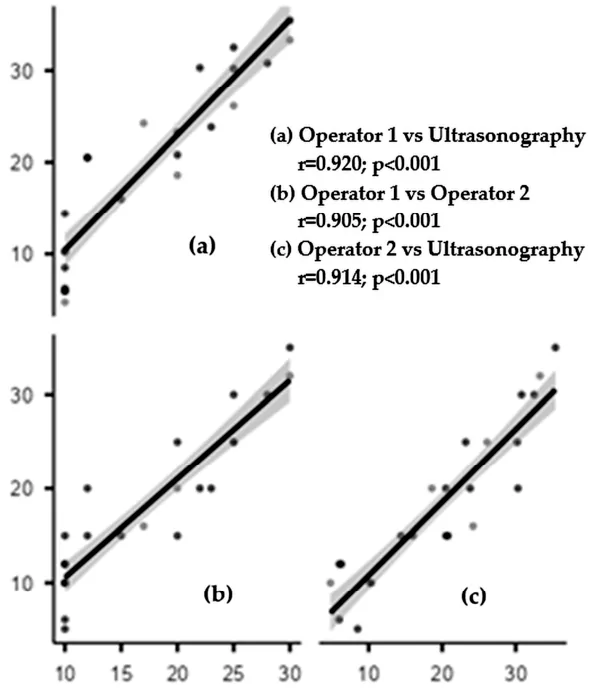
Scatter plots and correlations (r-value and *p*-value) between Operator 1 and ultrasound (**a**), Operator 1 and Operator 2 (**b**), Operator 2 and ultrasound (**c**).

**Figure 6:**
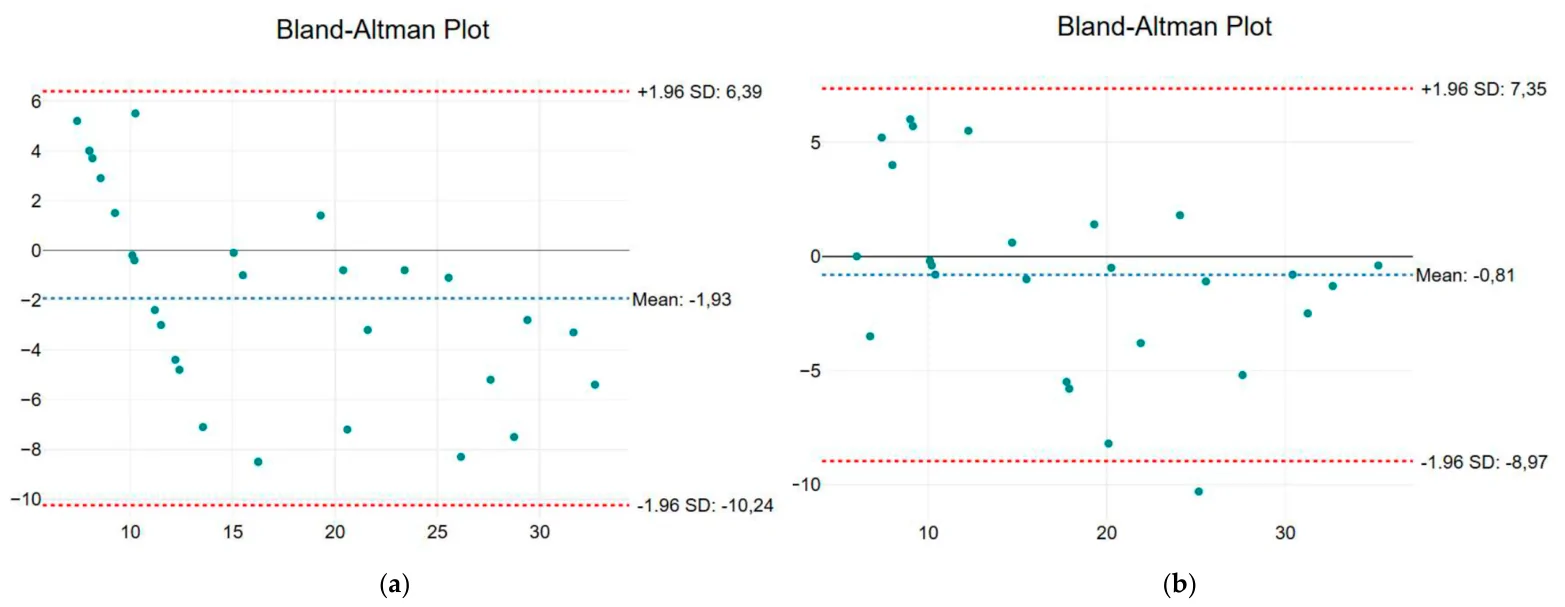
Bland–Altman plots for follicular diameters estimated by Operator 1 (**a**) or Operator 2 (**b**) and measured by ultrasound on 30 occasions.

A significant correlation was observed between both operators and ultrasound measurements, as well as between the two operators (Figure 5). However, palpation showed a 25% false-negative rate for detecting follicles ≥20 mm. Sensitivity and specificity were 75% and 90%, respectively, for both operators. Bland–Altman analysis showed acceptable agreement for Operator 1, while one outlier was observed for Operator 2. However, the limits of agreement (~8 mm) were relatively wide from a clinical perspective (Figure 6).

## 4. Discussion

The exotic pet market in North America and Europe has grown steadily in recent years, reaching an estimated value of USD 1.30 billion in 2023, with a corresponding increase in demand for specialized veterinary services [18,19]. Reptiles are expected to experience the fastest growth among exotic pets, with a compound annual growth rate of approximately 8% [19]. Despite this trend, scientific knowledge on snake reproduction, including that of the ball python, remains limited. Therefore, further studies providing baseline data are essential for improving captive breeding management.

In the wild, ball python reproduction is not easy to follow due to their living habits, including hiding in burrows most of the day, which prevents observation [20]. They appear to reach reproductive maturity relatively early and to remain reproductively active for many years, as females reach reproductive maturity from 27 to 31 months and males from 16 to 18 months and their lifespan in the wild is typically 10 years, although in captivity they can live far longer [20,21]. In their native environment, mating occurs in November–December, egg laying occurs from mid-January to the end of March and egg hatching occurs in May–June [20]. Females lay clutches of 4–15 eggs, depending on location: clutches in Ghana are larger (6–15 eggs) than those described in Benin and Togo (4–8 eggs) [20]. Earlier descriptions were similar, indicating clutches of 6–10 eggs laid from mid-February to the beginning of April [22]. The mean clutch size of wild-caught females was 7.7 eggs [23]. For around two months, after oviposition, females are brooding. During this period females remain tightly coiled around the eggs for the entire incubation period, without feeding, a cost to be added to the energy expended in shivering thermogenesis needed to maintain the correct temperature [23]. For these reasons, in captive Ball python breeding, artificial incubation, generally in custom incubators, is most often used [15].

In this study, ovulation and egg laying occurred in 58% of females. As expected, due to the very similar management, the two breeding farms showed similar outcomes. Although comparable data are scarce, large-scale breeder datasets suggest a reproductive frequency of approximately once every 1.97 years in ball pythons, consistent with our findings [18]. Similar reproductive intervals have been reported in other python species [24,25], whereas higher reproductive frequencies have been observed in wild ball pythons [26]. Improved understanding of follicular dynamics and closer monitoring may help identify factors influencing reproductive efficiency in captivity.

In the wild, ball pythons are monoestrous, with egg laying typically occurring between February and early April and hatching before the onset of the rainy season in May [2,21,23]. In captivity, reproductive timing appears to be considerably more variable. In this study, egg laying occurred between June and November, suggesting a reduction in seasonal reproductive synchrony, likely influenced by environmental factors such as temperature and photoperiod. The results from Morril’s (2011) thesis suggest that female ball pythons in captivity ovulate in all months of the year [5,18]. This plasticity could potentially be better exploited to optimize breeding management.

The management and husbandry employed in BF-1 and BF-2 (housing females in 80 × 60 × 20 cm plastic containers, each equipped with a heating mat providing a constant temperature of 32 °C at the warm spot, with a cooler temperature cycle of 26 °C from 20:00 to 08:00 before ovulation, relative humidity at 80%, deresinated spruce or dust-free beech shavings as substrate, rat diet) are within what is generally recommended or described for this species [5,7,8,15,27,28], despite slight variations in temperatures and/or humidity protocols. The rationale for also presenting results separately for each BF is to allow appreciation of possible variable outcomes, possibly due to the low number of animals per facility, despite the similar management and the same veterinarian responsible for their evaluation and health. Overall, respondents to the survey published by [15] manage a median of 45 animals, including males, females and youngsters, which is comparable to the size of the facilities described in this study. Therefore, the data presented can be considered representative of the conditions commonly encountered among hobby or small-scale breeders. This is different from the situation in large breeding farms like those described in other studies [8,18]. To our knowledge, no studies on this species were designed to compare different management strategies and environmental conditions for their effects on reproductive efficiency.

The mean ovulation-to-laying interval observed in BF-1 (50 days) was consistent with previous reports [5], whereas a mean shorter interval (45 days) was observed in BF-2. In this last facility, the interval was below 40 days on three occasions, including an interval of only 35 days for a clutch consisting entirely of slugs. These variations are not exceptional, considering that in the description of reproductive events in a large breeding farm the observed range was 37–78 days, based on 155 animals, distributed around a mean of 51 days [18]. In this study, due to the small number of animals, twenty-two, for which the ovulation to laying interval was recorded, individual animals may have had a significant effect. A longer mean interval (59 ± 10 days) between mid-body swelling and deposition was described for a small population of four females followed for two seasons [7]. Overall, variability in this interval is well documented [5,7,10] and may depend on individual, methodological or environmental factors. Individual or inherited effects on this interval have not been described; however, Morril (2011) observed significant heritability values for other reproductive traits: these were high for clutch mass and size (0.60 and 0.44), while lower for hatching rate (0.28) [18].

Ovulation was identified by the observation of body swelling in the caudal third and the perception of a watery mass upon palpation, as described by others [16,17]. However, it was also suggested that ovulation may occur earlier than at mid-body swelling [7]. According to [7], midbody swelling occurred at a time when the largest follicles were approximately 40–45 mm in diameter. After midbody swelling, mineralization and eggshell formation were identified as a thin hyperechoic rim surrounding the shed follicle. They concluded that this event was associated with eggshell development, not necessarily ovulation. However, in a later study on Boa constrictor, the coincidence between swelling and ovulation was confirmed in this species, in which the phenomenon was recognized in several subjects [17]. Improved methods for identifying ovulation in ball pythons could enhance accuracy in future studies.

In this study, clutch size (median: 6 eggs) was similar to or slightly lower than previously reported values [5,8,16,23], possibly reflecting differences in age, size, or management conditions. Incubation duration averaged approximately 58 days, in agreement with previous reports under similar temperature conditions [5]. Deviations from the average incubation period, such as the shorter duration observed in one clutch, may reflect individual or environmental variability. Earlier studies have demonstrated the strong influence of incubation temperature on development and survival [29]. The number of live hatchlings and proportion of infertile eggs in the present study were generally consistent with published data [5,23].

Ultrasonography proved to be a reliable technique for monitoring follicular development, confirming previous findings [6,7]. Follicular growth patterns observed in this study were consistent with classifications of growing, quiescent, and regressing follicles [7,8]. Notably, follicles that had not increased in size early in the season (March-April) did not subsequently ovulate. However, of the 28 females that ovulated during the study, four decreased in size of the largest follicle between the first and the second ultrasound session (−0.3, −1.2, −1.4, −1.5 mm). They could be considered stable, rather than regressing, and such small variations may lie in the possible intraobserver variability described by [8]. Moreover, all four females showed an increase in the largest follicle diameter at the subsequent evaluation. Thus, although we cannot exclude the possibility of a retarded follicular development in some individuals, early ultrasonographic evaluation may help predict reproductive outcomes, or an early identification of factors negatively affecting follicular growth. Early evaluation of follicular growth could also help optimize the timing of introducing the male to the female snake, maximizing the likelihood of a successful breeding event [28].

In females that subsequently ovulated, follicles measuring ≥20 mm showed a median daily growth rate of 0.38 mm/day. The higher growth rate observed in this study compared with previous reports likely reflects the fact that only follicles that had already exceeded 20 mm and subsequently ovulated were considered, corresponding to the advanced preovulatory phase rather than the earlier stages of follicular development. In a previous study mean follicular growth was 0.110 ± 0.071 mm/day between the end of the cooling period and pairing, roughly from 5 to 10 mm in diameter [8]. Knowledge of the follicular growth rate during the advanced preovulatory period may help optimize the timing of follow-up ultrasonographic examinations, thereby supporting reproductive management and reducing the likelihood of missing ovulation.

Another interesting application of ultrasonography would be predicting the time of ovulation. The ROC analysis identified an optimal follicular diameter threshold close to 31 mm for predicting ovulation within 30 days. However, ovulation dates were unavailable for six of the 28 ovulating females, and intra-observer repeatability of follicular measurements was not assessed. The ROC analysis could thus include only the 22 females for which an ovulation date was recorded, and it assessed the ability of follicular diameter to discriminate between ovulation within versus more than 30 days, rather than the overall probability of ovulation in all monitored females. Finally, the confidence intervals are wide, and it is unknown whether exposing females to males when follicles reached ~20 mm may have had an effect. While the identified absolute threshold value should thus be interpreted cautiously, it provides a practical ultrasonographic reference that may assist clinicians and breeders in estimating the proximity of ovulation. Future studies including larger populations are warranted to validate this threshold and determine its applicability across different breeding populations.

Finally, only 6.5% of breeders responding to a survey use ultrasound on females to observe follicular development [15]. Thus, evidently, coelomic palpation remains widely used despite possible risks for the female [8] and although its limitations were evident, with a substantial rate of false-negative results. Therefore, ultrasonography appears to be a more accurate tool for assessing reproductive status and optimizing breeding management.

## 5. Conclusions

Ultrasonography is a valuable and minimally invasive tool for reproductive monitoring in ball pythons, providing a more accurate assessment of follicular development than manual palpation. Monitoring follicular dynamics may improve breeding management by estimating the proximity of ovulation and helping optimize the timing of reproductive management decisions. The reproductive and ultrasonographic data in this study may also serve as a reference for future research and the development of reproductive biotechnologies, with potential benefits for captive husbandry, animal welfare, and conservation.

## Figures and Tables

**Table 1:** Ovulation to egg-laying time, number of eggs laid, proportion of “slugs”, laying–hatching interval, and live newborns, overall and by breeding farm (BF-1 and BF-2). Mean ± standard deviation or median (Q1–Q3), depending on data distribution, and number of animals (*n*).

	Overall	BF-1	BF-2
Ovulation to egg laying (days)	47.1 ± 6.1 (*n* = 22)	50.5 ± 5.4 ^A^ (*n* = 8)	45.1 ± 5.8 ^B^ (*n* = 14)
Eggs laid per subject	6 (5–7) (*n* = 28)	5 (5–6) ^A^ (*n* = 9)	6 (5–7) ^A^ (*n* = 19)
Number and proportion of “slug” eggs	0 (0–1) 0% (0–17) (*n* = 28)	0 (0–0) 0% (0–0) ^A^ (*n* = 9)	0 (0–2.2) 0% (0–42.5) ^B^ (*n* = 19)
Interval egg laying to hatching (days)	58 (57–59) (*n* = 24)	58.5 (58–59) ^A^ (*n* = 8 *)	58 (57–58.3) ^A^ (*n* = 16)
Live-born neonates/female	5 (4–7) (*n* = 27)	5 (5–6) ^A^ (*n* = 9)	5.5 (3–7) ^A^ (*n* = 18)

* in one case the exact day of hatching was not known. Different superscript letters within a row denote statistically significant differences. A ≠ B by *p* < 0.05.

**Table 2:** Largest follicular diameters observed in the six ultrasound sessions performed in females who ovulated during the study and those who did not. Median, Minimum and Maximum diameter and number of subjects (*n*).

Ultrasound Date	Females that Ovulated During the Study			Females that did not Ovulate During the Study	
	*n* Animals Examined	*n* Already Ovulated	Median Follicular Diameter (Minimum, Maximum) (mm)	*n* Animals Examined	Median Follicular Diameter (Minimum, Maximum) (mm)
2 March	28	0	15.10 ^a^ (7.3–26.1)	20	9.40 ^b^ (4.8–16.2)
23 March	28	0	20.90 ^a^ (10.5–34.4)	20	10.10 ^b^ (5.7–19.1)
29 April	18	10	25.10 ^a^ (14.0–39.0)	20	10.40 ^b^ (5.5–18.4)
1 June	11	17	32.0 ^a^ (20.0–39.3)	20	11.7 ^b^ (5.0–20.0)
20 July	1 *	26	23.0	18	9.75 (5.0–22.7)
18 August	1	27	25.0	20	0.0 (0.0 **−21.7)

* One subject, who ovulated on 8 August, did not undergo ultrasound. ** In 13 subjects, there were no measurable follicles, and the value was recorded as 0 mm. Within the same date: a ≠ b (*p* < 0.05).

## Data Availability

The data presented in this study are available on request from the corresponding author due to the inclusion of privately owned animals.

## Abbreviations

The following abbreviations are used in this manuscript:

BF-1	Breeding facility 1
BF-2	Breeding facility 2
